# Tetralogy of Fallot - Born to be Bad?

**DOI:** 10.1186/1749-8090-10-S1-A194

**Published:** 2015-12-16

**Authors:** Safak Alpat, Sevgen Ç Önder, Mustafa F Sargon, Murat Güvener, Mustafa Yılmaz, Rıza Doğan, Metin Demircin, İlhan Paşaoğlu

**Affiliations:** 1 Department of Cardiovascular Surgery, Hacettepe University School of Medicine, Ankara, 06100, Turkey; 2 Department of Pathology, Hacettepe University School of Medicine, Ankara, 06100, Turkey; 3 Department of Anatomy, Hacettepe University School of Medicine, Ankara, 06100, Turkey

## Background/Introduction

TOF repair can be performed with low morbidity and mortality in many centers. However, some patients can experience a prolonged, troublesome postoperative recovery, associated with higher inotopic scores, extended duration of ventilation, longer intensive care and hospital stays. Underlying pathophysiologic explanation for this concept can be defined as combined myocyte injury and fibrosis in right ventricle of patients with TOF that occurred in response to chronic hypoxia and volume overload, even before corrective surgery.

## Aims/Objectives

In this study, we analyzed myocyte injury and fibrosis with histopathological and ultrastructural methods in right ventricle outflow tract muscle tissues that obtained from 14 patients with TOF during corrective surgery. Our aim was to determine correlation between these alterations and early surgical outcomes.

## Method

Fourteen patients with TOF who underwent surgical repair were recruited. Patients were divided into the cyanotic (SO2>90%, n = 7) and noncyanotic groups (SO2<90%, n = 7). Resection of RV infundibular muscles was performed during standard repair. Myocyte injury and fibrosis were analysed via histopathologically and ultrastructurally. Pre-, intra and postoperative characteristics of patients were recorded.

## Results

There were no significant differences between the group with regard to pre- and intraoperative variables (p > 0.05). There was no mortality. However, inotropic score, inotropic therapy duration, intensive care stay and in-hospital stay were found significantly higher in cyanotic group (p < 0.05). Histopathologic examination revealed that myocyte injury and ultrastructurally defined mitochondria injury score were also higher in cyanotic patients (p = 0.01, p = 0.02). Despite all patients showed some degree of fibrosis in their specimens, cyanotic patients had more severe fibrosis than non-cyanotic patients (p = 0.02). Nonetheless, we found no correlation between histopathological alterations and early surgical outcomes (p > 0.05). Incidentally, we detected positive correlation in between preoperative history of spell and poor early surgical outcomes (p < 0.05).

## Discussion/Conclusion

As a result, we showed that myocyte injury and fibrosis had occurred even before TOF repair and cyanotic patients had more severe myocyte injury and fibrosis. Nevertheless, these alterations were not in correlation with early surgical outcomes. Furthermore, history of spell was positively correlated with poor early surgical outcomes.

